# Recent Developments in Data-Assisted Modeling of Flexible Proteins

**DOI:** 10.3389/fmolb.2021.765562

**Published:** 2021-12-24

**Authors:** Cezary Czaplewski, Zhou Gong, Emilia A. Lubecka, Kai Xue, Chun Tang, Adam Liwo

**Affiliations:** ^1^ Faculty of Chemistry, University of Gdańsk, Gdańsk, Poland; ^2^ Innovation Academy for Precision Measurement Science and Technology, Chinese Academy of Sciences, Wuhan, China; ^3^ Faculty of Electronics, Telecommunications and Informatics, Gdańsk University of Technology, Gdańsk, Poland; ^4^ PKU-Tsinghua Center for Life Sciences, Beijing National Laboratory for Molecular Sciences, College of Chemistry and Molecular Engineering, Peking University, Beijing, China

**Keywords:** proteins, data-assisted modeling, conformational ensembles, nuclear magnetic resonance, small-angle X-ray scattering, chemical cross-linking coupled with mass spectroscopy, molecular dynamics, coarse graining

## Abstract

Many proteins can fold into well-defined conformations. However, intrinsically-disordered proteins (IDPs) do not possess a defined structure. Moreover, folded multi-domain proteins often digress into alternative conformations. Collectively, the conformational dynamics enables these proteins to fulfill specific functions. Thus, most experimental observables are averaged over the conformations that constitute an ensemble. In this article, we review the recent developments in the concept and methods for the determination of the dynamic structures of flexible peptides and proteins. In particular, we describe ways to extract information from nuclear magnetic resonance small-angle X-ray scattering (SAXS), and chemical cross-linking coupled with mass spectroscopy (XL-MS) measurements. All these techniques can be used to obtain ensemble-averaged restraints or to re-weight the simulated conformational ensembles.

## 1 Introduction

Proteins exist as dynamic structures. Many proteins undergo often very significant motions while performing their functions (Henzler-Wildman and Kern, 2007; Boehr et al., 2009). The respective conformational states are sometimes stable enough to be captured through X-ray structure determination if appropriate conditions of protein-sample preparation are applied (Bertelsen et al., 2009; Kityk et al., 2012). Nevertheless, in most instances, the structures of multistate proteins, as well as those of intrinsically disordered proteins (IDPs) or proteins with intrinsically-disordered regions (IDRs) can be described only in terms of conformational ensembles. Over 40% of human proteins contain stretches of disorder longer than 30 residues (van der Lee et al., 2014).

**FIGURE 1:**
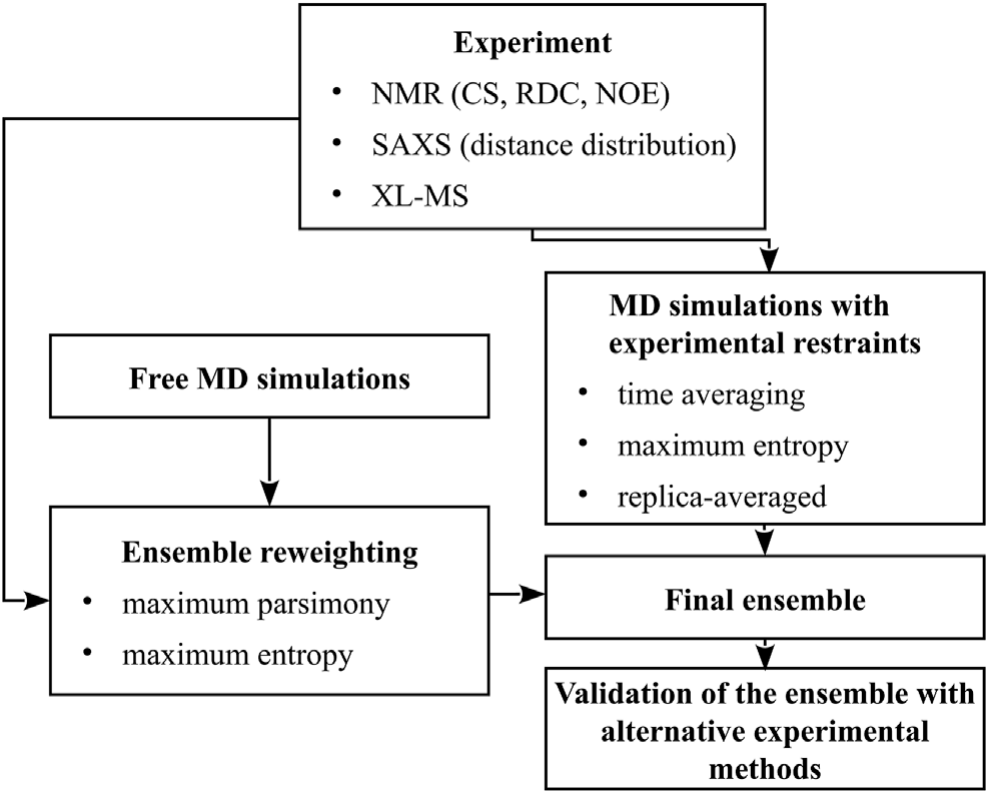
A scheme of methods for the determination of conformational ensembles of flexible proteins.

Thus, ensemble-averaged quantities are usually obtained from measurements while studying conformational dynamics of multistate proteins, IDPs, or flexible peptides. The composition of an ensemble can be determined only by combining the results of measurements with advanced molecular modeling (Bonomi et al., 2017; Bonomi and Vendruscolo, 2019; Orioli et al., 2020). In this minireview, we summarize the methods for conformational-ensemble determination using molecular modeling, using the data from nuclear magnetic resonance (NMR), small-angle X-ray scattering (SAXS), and chemical cross-linking coupled with mass spectroscopy (XL-MS). In Section 2, we outline the experimental techniques mentioned above and the quantities that they provide, while in Section 3 we describe conformational-sampling methods and two major approaches of implementing the experimental quantities in conformational-ensemble determination: simulations with ensemble-averaged restraints and ensemble reweighting. A scheme summarizing the methodologies discussed is shown in Figure 1.

## 2 Experimental Methods to Study Flexible Proteins

Here we focus on the experimental measurements that can be performed for proteins in solution. We leave out the single-molecule fluorescence resonance energy transfer (FRET), which does not yield ensemble averages and does not have the same issues as those discussed in Section 3 (Tang and Gong, 2020; Lerner et al., 2021).

### 2.1 Nuclear Magnetic Resonance

The most complete information about the structure and conformational dynamics of proteins and peptides is provided by NMR (Sekhar and Kay, 2019). NMR remains the method of choice to characterize the conformational dynamics of proteins to atomic resolution in near-physiological conditions. NMR observables, including nuclear Overhauser effect (NOE), chemical shift, dipolar coupling constants, and paramagnetic relaxation enhancement (PRE) are ensemble-averaged over a multitude of conformational states (Salmon et al., 2010; Konrat, 2014; Clore, 2015; Huang et al., 2015; Tang and Gong, 2020). Thus, though the flexible regions in a protein can be easily identified by NMR owing to their favorable relaxation properties, it is difficult to obtain a comprehensive description of the ensemble structure of a multi-domain protein or an IDP as a whole and determine the fractions of the constituting conformational states. To this end, many methods have been developed to reconstruct the ensembles based on the NMR data (Bertini et al., 2004; Mittag and Forman-Kay, 2007; Delaforge et al., 2015).

Paramagnetic NMR, in particular, paramagnetic relaxation enhancement (PRE), allows the visualization of protein ensemble structures (Otting, 2010; Liu et al., 2016). The PRE is exquisitely sensitive to the sparsely populated conformations, thanks to the large gyromagnetic ratio of an unpaired electron in the paramagnetic probe and an inverse sixth power dependence on the distances to the observed NMR nuclei (Clore, 2015; Liu et al., 2015). On the other hand, covalent attachment of a paramagnetic probe could perturb the structure, which is more likely for an IDP (Sasmal et al., 2017). As a result, paramagnetic cosolute molecules have been developed (Gu et al., 2014; Gong et al., 2017a), which can also be used to assess the dynamic structures of IDPs (Hartlmüller et al., 2019; Spreitzer et al., 2020). Similar to the PREs, the NOEs also provide ensemble-averaged distances between protein nuclei. However, quantitative interpretation of the NOEs is hampered by the complex relaxation pathways. The exact proton-proton distances and the corresponding conformational states of a protein are best extracted on a perdeuterated background (Vögeli et al., 2009; Vögeli et al., 2016).

### 2.2 Small-Angle Scattering Methods

Compared to NMR, small-angle X-ray and small-angle neutron scattering (SANS) provide less detailed but more global structural information (Konarev et al., 2003; Forster et al., 2008; Schneidman-Duhovny et al., 2011; Trewhella et al., 2013). For a multi-state protein, the scattering curve is averaged over a multitude of conformational states. The different states and the associated population can, in theory, be obtained from the deconvolution of the scattering curve. To this end, many algorithms have been developed that include ensemble optimization method (EOM) (Bernadó et al., 2007; Tria et al., 2015), minimal ensemble search (MES) (Pelikan et al., 2009), and Bayesian ensemble SAXS (BE-SAXS) (Antonov et al., 2016). Though the scattering intensity at each scattering angle is normally used as a restraint (Forster et al., 2008), pairwise distance distribution could also be employed for the comparison between different sets of structure ensembles (Gorba et al., 2008; Karczyńska et al., 2018). The different approaches fit different numbers of parameters and use different treatments of the displaced solvent, which inevitably leads to somewhat different solutions.

### 2.3 Chemical Cross-Linking Coupled With Mass Spectroscopy

Cross-linking reactions are initiated either by illumination or chemical reaction followed by enzymatic digestion. The final products are cross-linked peptides, which can be identified by mass spectrometry with high confidence. The cross-linked residues have to be closer in distance than the length of the cross-linker arm. Therefore, each cross-link can be used to derive the restraint imposed on the C^*α*^ … , C^*α*^-, C^*β*^ … , C^*β*^- or the terminal-atom (e.g., N^*ζ*^ … , N^*ζ*^ atom pair of lysine side chains) distance of the two cross-linked residues. However, the cross-links may artificially pull two protein regions together, in a so-called zippering effect (Belsom and Rappsilber, 2021), which needs to be carefully controlled and ruled out.

The identified cross-links are often found incompatible with the known protein structure, in which the calculated distance exceeds the maximum length of the cross-linker. Such “over-length” cross-links can be explained by alternative protein conformations, e.g., an open-to-closed transition (Ding et al., 2017), or by the transient oligomerization of the protein. The latter can be ascertained with the mixing of “light” and “heavy” proteins with distinct isotope labeling patterns (Gong et al., 2015). Furthermore, cross-linking mass spectrometry (XL-MS) can be used to elucidate dynamic encounters between two proteins (Gong et al., 2017b).

A crosslink restraint is usually imposed on the straight-line distance between the C^*α*^-atoms of the corresponding residues (Leitner et al., 2014; Merkley et al., 2014; Fajardo et al., 2019). Recently, we developed an approach in which restraints are imposed on side-chain ends and implemented it in all-atom (Gong et al., 2020) and coarse-grained (Kogut et al., 2021) molecular dynamics. This approach is more realistic because such distances are close to those between the solvent-accessible surfaces, which are targeted by the cross-linking reagents in the XL-MS experiments.

## 3 Modeling Protein Structures With Experimental Restraints

### 3.1 Conformational Search

Canonical molecular dynamics (MD) (Frenkel and Smit, 2000) and its extensions, namely simulated annealing (SA) (Kirkpatrick et al., 1983), replica-exchange molecular dynamics (REMD) (Hansmann, 1997), and multiplexed replica exchange molecular dynamics (MREMD) (Rhee and Pande, 2003) are usually the methods of choice for sampling the conformational space, owing to their efficiency. All-atom MD is commonly used and a variety of good algorithms and software packages such as e.g., AMBER (Salomon-Ferrer et al., 2013), CHARMM (Brooks et al., 2009), GROMACS (Abraham et al., 2015), LAMMPS (Plimpton, 1995) and DESMOND (Bowers et al., 2006) are available, which also enable the researchers to include experimental information as restraints.

All-atom MD has restricted ability to sample the conformational space extensively (Bottaro and Lindorff-Larsen, 2018). Compared to all-atom approaches, the coarse-grained (CG) approaches, in which several atoms are merged into extended interaction sites, are computationally more efficient and enable us to run simulations at much longer time-scales and for larger systems (Voth, 2008; Kmiecik et al., 2016). The coarse-grained models with which MD for proteins can be run include MARTINI (Marrink and Tieleman, 2013), AWSEM (Davtyan et al., 2012), OPEP (Sterpone et al., 2014), and UNRES (Liwo et al., 2019). CABS (Kolinski, 2004) is another very good CG model of proteins, which was developed to run Monte Carlo dynamics on a high-resolution lattice.

The experimental information can be used as restraints or to filter the conformational ensembles/reweight its conformations to reproduce the experimental observables (Bonomi et al., 2017; Orioli et al., 2020). These two approaches are described in the two subsequent subsections.

### 3.2 Restrained Simulations of Conformationally Heterogeneous Systems

In restrained simulations, penalty terms are added to the potential energy in MD so that the forces consist of the forces computed from the force field of choice and those due to restraint violation (van Gunsteren et al., 2016). This approach is straightforward if a protein has a well-defined structure and has been implemented in the CYANA (Güntert and Buchner, 2015) and XPLOR-NIH software packages (Schwieters et al., 2018) for structure determination by NMR, as well as is built in the MD packages mentioned in the previous section. For flexible systems, time- and ensemble averaging algorithms to run restrained simulations have been developed.

It should be noted that using restraints from NMR in CG simulations is not straightforward, because the respective quantities depend on all-atom geometry. One method, in which the CG structures are converted into all-atom structures, from which the respective quantities are calculated, was developed (Latek and Koliński, 2011) for use with the CABS model of proteins (Kolinski, 2004). However, this method is not suitable for restrained MD simulations, because it does not provide the forces due to restraints. Recently, we developed ESCASA (Lubecka and Liwo, 2021), an analytical approach to calculating approximate positions of the protons from C^*α*^-trace geometry, thus enabling us to compute the forces due to the penalty function and, consequently, to use the method with coarse-grained MD.

#### 3.2.1 Time-Averaged Restraints

In the time-averaged-restraint method, the quantities obtained from simulations (e.g., interproton distances) are averaged over a time window (Torda et al., 1989; Bonvin et al., 1994). These average quantities are inserted into the penalty terms.

$$\bar{f}\left(r\left(t\right)\right)={\left[\tau \left(1-\exp\left(-t/\tau \right)\right)\right]}^{-1}\overset{t}{\underset{0}{\int }}\exp\left(-t^{\prime }/\tau \right)f\left(r\left(t-t^{\prime }\right)\right)dt^{\prime }$$ (1)

where *f* is the quantity being averaged, which depends on the coordinates of the atoms of the system contained in vector **r** and *τ* is the length of the time window.

#### 3.2.2 Ensemble Averaged Restraints

The methods that use ensemble-averaged restraints are based on the maximum-entropy and Bayesian principles, according to which a minimally perturbed conformational ensemble compared to that resulting from free simulations is sought and, at the same time, the ensemble-average quantities match their experimental counterparts within the experimental error (Pitera and Chodera, 2012; White et al., 2015; Amirkulova and While, 2019). If the ensemble-averaged restraints are enforced strictly, the potential-energy function is modified to include the experimental quantities with the weight calculated to maximize the entropy (Pitera and Chodera, 2012).

$$U_{ME}\left(r;\alpha_{1},\ldots ,\alpha_{M}\right)=U\left(r\right)+\overset{N}{\underset{i=1}{\sum }}\alpha_{i}f_{i}\left(r\right)$$ (2)

where *f*_*i*_(**r**) is the value of the *i*th experimental observable calculated for the conformation described by the vector of coordinates **r**, *N* is the number of observables, *U* is the potential-energy function used in MD simulations, *U*_*ME*_ is the extended energy function and the weights *α*_*i*_ are calculated to minimize Γ(*α*_1_, … , *α*_*N*_).

$$\Gamma \left(\alpha_{1},\ldots ,\alpha_{N}\right)=\ln\int \exp\left[-\beta U_{ME}\left(r;\alpha_{1}\ldots ,\alpha_{N}\right)\right]d^{3n}r-\beta \overset{N}{\underset{i=1}{\sum }}\alpha_{i}f_{i,\exp}$$ (3)

where *f*_*i*, *exp*_ is the experimental (ensemble-averaged) value of the *i*th observable, *β* = 1/*RT*, *R* being the universal gas constant and *T* absolute temperature, and *n* is the number of atoms in the system. It should be noted that the integral in Equation 3 does not have to be evaluated, because minimization of Γ leads to equations which contain the observables averaged over the conformations, which can readily be calculated from MD simulations (Pitera and Chodera, 2012). With this approach, the distribution of conformations is minimally perturbed with respect to that resulting from the force field used. In other words, the experimental constraints enable us to compensate for the inevitable inaccuracy of the force field and to obtain a distribution of conformations in the ensemble, which is closer to the true (Boltzmann) distribution (Cavalli et al., 2013), provided that the experimental data are sufficient in number and quality. In practical implementation, the replica-averaged method is applied (Camilloni et al., 2013; Hummer and Köfinger, 2015), in which several replicas are run with the extended potential energy, *U*_*ED*_, containing harmonic restraints on the experimentally measured quantities that are averaged over all replicas.

$$U_{ED}\left(r_{i}\right)=U\left(r_{i}\right)+M\underset{j}{\sum }\frac{{\left(\frac{1}{M}\overset{M}{\underset{k=1}{\sum }}f_{j}\left(r_{k}\right)-f_{\exp,j}\right)}^{2}}{2\sigma_{j}^{2}}$$ (4)

where the index *i* runs over replicas, *M* is the number of replicas, **r**_*k*_ is the vector of the coordinates of the conformation of the *k*th replica, and *σ*_*j*_ is the error in the *j*th observable. It has been demonstrated that this method becomes the maximum-entropy method as the number of replicas increases (Pitera and Chodera, 2012; Cavalli et al., 2013; Roux and Weare, 2013; Hummer and Köfinger, 2015). This approach has been implemented in determining the conformational ensembles from NMR (Camilloni et al., 2013) and SAXS data (Hermann and Hub, 2019). A similar approach termed dynamic ensemble refinement (DER) (Lindorff-Larsen et al., 2005) was developed earlier for the determination of protein dynamical ensembles from NMR data.

### 3.3 Reweighting the Conformational Ensembles

In the ensemble-reweighting methods, a pool of conformations is generated first in unrestrained simulations and, subsequently, the weights of the conformations are determined to reach the agreement of the conformation-averaged observables with the corresponding experimental quantities (Cavalli et al., 2013; Orioli et al., 2020). An advantage of this approach is that the ensemble can be generated once and used as the results of new experiments are available. However, the state-of-the-art force fields do not produce the true Boltzmann distribution of the conformational states. Consequently, the distribution of conformations obtained in unrestrained simulations could be far from the true distribution; specifically, some regions of conformational space that are, in reality, visited by the system might happen to be under-represented or omitted from the simulated ensemble. It has been demonstrated (Ceriotti et al., 2012) that the more the input distribution diverges from the true distribution the greater the error in reweighting. When the experimental information is included in the simulations as maximum-entropy constraints or replica-averaged restraints, the ensemble is driven towards reproducing the experimental data, i.e., closer to the true (unknown) Boltzmann distribution. An example that the quality of the force field becomes less important with increasing the number of data is the work by Joo et al. (Joo et al., 2015), in which a force field that contained only the van der Waals repulsion, stereochemistry, improper-torsion, and chirality terms, in combination with NOE and dihedral-angle restraints, was used with success to determine protein structures from NMR data.

Because the number of conformations in the ensemble (and, thereby, the number of weights) is usually much greater than the number of observables, the fitting problem is underdetermined. It is solved by using either the maximum-parsimony or the maximum-entropy principle (Bonomi et al., 2017).

In the maximum-parsimony approaches, a minimum set of conformations is determined that can reproduce the experimental observables. This method was originated by Nikiforovich and coworkers (Nikiforovich et al., 1987) and, subsequently evolved into a variety of algorithms, including EOM (Bernadó et al., 2007), ASTEROIDS (Nodet et al., 2009), and SES (Berlin et al., 2013), as well as the algorithms developed in our laboratories to determine the conformational ensembles from the SAXS (Kozak et al., 2010) or SAXS, NMR and XL-MS data (Liu et al., 2018). Usually, the ensemble is clustered first and averages are computed over each cluster, the weights of the clusters being determined by least-square fitting the ensemble-averaged observables to the experimental quantities, subject to the condition that all weights are non-zero and the number of clusters with non-zero weights is minimal.

In the maximum-entropy approach, the weights of conformations are determined so that the ensemble-averaged quantities match the experimental counterparts with minimal perturbation of the input ensembles. Usually, the experimental errors are included in the target function, which results in solving a Bayesian problem, with the prior distribution being equal to that from unrestrained MD simulations.

$$\theta \overset{M}{\underset{j=1}{\sum }}w_{j}\ln w_{j}+\overset{N}{\underset{i=1}{\sum }}\frac{{\left(\sum_{j=1}^{M}w_{j}f_{ij}-f_{i,\exp}\right)}^{2}}{2\sigma_{i}^{2}}=\min$$ (5)

where the first term is the negative of the Shannon entropy, *θ* being the weight of this term, and the weights are required to be normalized to unity and non-negative. Many approaches that use this principle, including ENSEMBLE (Marsh and Forman-Kay, 2012; Krzeminski et al., 2013), EROS (Różycki et al., 2011), COPER (Leung et al., 2016), and others (Groth et al., 1999) were developed.

Recently, Pesce and Lindorff-Larsen (Pesce and Lindorff-Larsen, 2021) designed an iterative maximum-entropy reweighting method for the determination of conformational ensembles from SAXS data, in which background intensity and the scaling factor of the computed average SAXS profile are fitted to match the experimental profile. Subsequently, the weights are determined by minimizing the target function of Equation 5. The two steps are iterated until convergence is achieved. The determination of background intensity and scaling factor is a major step forward with respect to the previous approaches, in which only the weights were determined, because these parameters depend on many features of the system studied (e.g., the solvation shell) and on experimental conditions. Also, very recently, an ensemble-reweighting method by using side-chain NMR-relaxation, termed Average Block Selection Using Relaxation Data with Entropy Restraint (ABSURDer), an extension of the ABSURD method of Blackledge and others (Salvi et al., 2016), has been developed by the Lindorff-Larsen group (Kümmerer et al., 2021). This approach takes into account system dynamics, thus enabling us to find the ensemble of trajectories, not just static conformations, consistent with experiment.

## 4 Conclusion and Outlook

Investigation of the dynamic structures of proteins and other biomolecules in solution is a rapidly growing field, in which the experimental and theoretical methods are complementary to each other (Bonomi et al., 2017; Bonomi and Vendruscolo, 2019; Orioli et al., 2020). Since the experiment provides only average observables (NMR), distance distribution (SAXS, SANS, and WAXS), or just indicates which residues may be close to each other in part of the dynamic structure (XL-MS), dynamic structure determination from the experiment alone is an underdetermined problem. Thus, the development of efficient and reliable conformational-search methods and better force fields is a necessity.

At present, the respective algorithms are based mostly on ensemble reweighting (Bonomi et al., 2017; Bonomi and Vendruscolo, 2019; Orioli et al., 2020), the maximum-entropy variant of which seems to be better, because it does not leave out any part of the ensemble completely, an important feature given the under-determinability of the reweighting problem (Bonomi et al., 2017). Because the conformational ensemble is generated in unrestrained simulations, this approach depends on the quality of a force field used, which is usually still far from being perfect. Therefore, the development of methods based on replica-averaged restraints, which stem from the maximum-entropy principle (Cavalli et al., 2013; Hummer and Köfinger, 2015) seems to be a better approach. Combining this approach with time-averaged restraints (Torda et al., 1989; Bonvin et al., 1994) or posterior ensemble fitting to enrich the averaging is recommended. An efficient conformational search is required regardless of choosing a particular method to include the experimental data, which can be carried out with coarse-grained models (Voth, 2008; Kmiecik et al., 2016). Deep-learning algorithms are also likely to advance the field, especially given their recent tremendous success in predicting the stable structures of proteins at crystallographic accuracy (Baek et al., 2021; Jumper et al., 2021). These methods may be used to generate the initial models for studying the dynamics of multistate proteins.

Another challenge is capturing the full dynamics of the system under study. Time-resolved techniques are an obvious answer here but averages, such as kinetic rate constants, can also be used – an approach has recently been proposed (Brotzakis et al., 2021). This will be particularly important when studying the dynamics of multistate proteins with more than two stable states.
